# Genomic signatures of near-extinction and rebirth of the crested ibis and other endangered bird species

**DOI:** 10.1186/s13059-014-0557-1

**Published:** 2014-12-11

**Authors:** Shengbin Li, Bo Li, Cheng Cheng, Zijun Xiong, Qingbo Liu, Jianghua Lai, Hannah V Carey, Qiong Zhang, Haibo Zheng, Shuguang Wei, Hongbo Zhang, Liao Chang, Shiping Liu, Shanxin Zhang, Bing Yu, Xiaofan Zeng, Yong Hou, Wenhui Nie, Youmin Guo, Teng Chen, Jiuqiang Han, Jian Wang, Jun Wang, Chen Chen, Jiankang Liu, Peter J Stambrook, Ming Xu, Guojie Zhang, M Thomas P Gilbert, Huanming Yang, Erich D Jarvis, Jun Yu, Jianqun Yan

**Affiliations:** Xi’an Jiaotong University, Xi’an, 710049 P. R. China; BGI-Shenzhen, Shenzhen, 518083 P. R. China; CAS Key Laboratory of Genome Sciences and Information, Beijing Institute of Genomics, Chinese Academy of Sciences, Beijing, 100101 P.R. China; Center for Mitochondrial Biology and Medicine, The Key Laboratory of Biomedical Information Engineering of Ministry of Education, School of Life Science and Technology and Frontier Institute of Science and Technology, Xi’an Jiaotong University, Xi’an, 710049 China; University of Wisconsin School of Veterinary Medicine, Madison, Wisconsin USA; BGI-Northwest, BGI-Shenzhen, Xi’an, 710000 P. R. China; Kunming Institute of Zoology, Chinese Academy of Sciences, Kunming, Yunnan P. R. China; James D. Watson Institute of Genome Science, Hangzhou, China; Department of Biology, University of Copenhagen, Copenhagen, Denmark; King Abdulaziz University, Jeddah, Saudi Arabia; School of Biomedical Sciences, University of Queensland, Queensland, Australia; University of Cincinnati College of Medicine, Cincinnati, Ohio USA; College of medicine, University of Chicago, Chicago, USA; Centre for GeoGenetics, Natural History Museum of Denmark, University of Copenhagen, ØsterVoldgade 5-7, 1350 Copenhagen, Denmark; Department of Environment and Agriculture, Trace and Environmental DNA Laboratory, Curtin University, Perth, Western Australia 6845 Australia; Prince Aljawhra Center of Excellence in Research of Hereditary Disorders, King Abdulaziz University, Jeddah, Saudi Arabia; Howard Hughes Medical Institute, Duke University Medical Center, Durham, NC 27710 USA

## Abstract

**Background:**

Nearly one-quarter of all avian species is either threatened or nearly threatened. Of these, 73 species are currently being rescued from going extinct in wildlife sanctuaries. One of the previously most critically-endangered is the crested ibis, *Nipponia nippon*. Once widespread across North-East Asia, by 1981 only seven individuals from two breeding pairs remained in the wild. The recovering crested ibis populations thus provide an excellent example for conservation genomics since every individual bird has been recruited for genomic and demographic studies.

**Results:**

Using high-quality genome sequences of multiple crested ibis individuals, its thriving co-habitant, the little egret, *Egretta garzetta*, and the recently sequenced genomes of 41 other avian species that are under various degrees of survival threats, including the bald eagle, we carry out comparative analyses for genomic signatures of near extinction events in association with environmental and behavioral attributes of species. We confirm that both loss of genetic diversity and enrichment of deleterious mutations of protein-coding genes contribute to the major genetic defects of the endangered species. We further identify that genetic inbreeding and loss-of-function genes in the crested ibis may all constitute genetic susceptibility to other factors including long-term climate change, over-hunting, and agrochemical overuse. We also establish a genome-wide DNA identification platform for molecular breeding and conservation practices, to facilitate sustainable recovery of endangered species.

**Conclusions:**

These findings demonstrate common genomic signatures of population decline across avian species and pave a way for further effort in saving endangered species and enhancing conservation genomic efforts.

**Electronic supplementary material:**

The online version of this article (doi:10.1186/s13059-014-0557-1) contains supplementary material, which is available to authorized users.

## Background

The International Union for Conservation of Nature (IUCN) and Bird Life Species has recognized over 20% of approximately 10,000 extant bird species as being threatened. As of 2014, the IUCN RedList has declaredfive, 1,373, and 959 species as extinct in the wild, threatened, and near threatened, respectively. Between 1988 and 2008, the conservation status of 235 species was upgraded to higher categories of endangerment, as compared to just 32 species that were downgraded [1]. Furthermore, historical records document the extinction of at least 150 avian species since the 16th century. The principal threats leading to avian population decline have been linked to man-made environmental disasters, including over-hunting, habitat loss, pesticide abuse, and invasive species introduction [2]. To combat the ongoing decline, conservation efforts have been made, such as protective legislation, habitat restoration, captive breeding, and reintroduction, and all are responsible for the successful recovery of 49 species that were near-extinct between 1994 and 2004 [3].

Recent conservation genetic studies [4-8] have demonstrated that small populations are susceptible to allelic drift, leading to allele loss/fixation, and the process can be accelerated by inbreeding. Likewise, in small captive populations, rapid genetic deterioration, such as inbreeding depression and genetic adaptation to artificial environment, can also occur [8]. Deleterious mutation tends to accumulate due to reduced selective strength [4]. Furthermore, extinction rate in small wild populations increases significantly as heterozygosity decreases [5]. Several genetic studies have attempted to characterize this effect from conservation-related bottlenecks among avian species, albeit based on limited markers of allozymes or microsatellites [9,10]. It has been proposed that studies using up-to-date and more informative markers at a genome-scale will be necessary [11].

**Figure 1:**
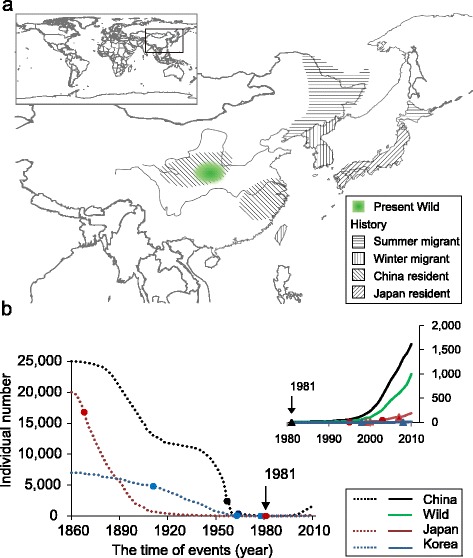
**Demographic history of the crested ibis and its population dynamics. (a)** The crested ibis populations (summer migrants, winter migrants, China residents, and Japan residents) were once widely distributed in East Asia. The recorded habitats are marked with parallel lines. The two breeding pairs were discovered in 1981 in the area in the South Qingling Mountains (green shade). **(b)** Population history based on historic records and the scientific literature [14]. The curves (dotted lines) indicate the time at which population bottlenecks occurred and bottleneck milestones are shown as solid diamonds (Additional file 1: Table S1). The inset enlarges the curves from 1980 to 2010. The colored solid triangles indicate recorded historic events (Additional file 1: Table S2). The vertical downward arrows indicate the discovery of the two survived breeding pairs in 1981.

One of the most recently endangered bird species in the world is the crested ibis (*Nipponia Nippon*; IUCN Red Data Book, BirdLife International 2001). It was once widespread across Northeast Asia, with a range encompassing China, Russia, Korea, and Japan until the 1860s (Figure 1a). Suffering from over-hunting and habitat loss, the crested ibis populations had finally collapsed in the late 19th and early 20th centuries, to the extent that it was thought to be completely extinct from the wild, when the last five birds were taken into captivity in Japan in 1981 [2] (Figure 1b; Additional file 1: Tables S1, S2). Although extensive private and government-supported efforts were made to revive the species in Japan, the final five individuals taken into captivity died without producing offspring. However, through an extensive international survey effort aimed at saving the species from extinction, a remarkable discovery was made later in 1981. After over 17 years of no sightings in China (since one loner bird seen in the wild in 1964), seven individuals belonging to two breeding couples (four adults, of which one couple had three chicks) were found at the edge of their normal habitats in a remote location in the southern foothills of the Qinling Mountains (Yangxian County, Shanxi). To protect these seven birds, China immediately established the Yangxian Nature Reserve, and a conservation program for both wild and captive birds was started. Monitor stations were set up for every nest, GPS-based systems for individual identification and tracking, and guidelines that control pesticides and habitat destruction were introduced [12-14]. As a result, the two breeding pairs became what can be considered the ‘Adam and Eve’ for the recovered crested ibis population that has gone through two phases in over 33 years: initially, a small size (<100) between 1981 to 2001, and up to over 2,000 individuals currently (Figure 1b).

To provide genome-scale insights into the near-extinction and rescue, we sequenced the genomes of multiple individuals from both the crested ibis (n = 9; from the rescued population) and its co-habitant, not-endangered close-relative, the little egret (*Egretta garzetta*; n = 6; from the same order *Pelecaniformes*; diverged approximately 57 million years ago) [15]. We compared their genome sequences with those of 41 other avian species described in companion publications in this issue and elsewhere [16], which include seven Endangered + Vulnerable (EV) species listed by the IUCN within the recent past (crowned crane, *Balearica regulorum*; macQueen’s bustard, *Chlamydotis macqueenii*; brown mesite, *Mesitornis unicolor*; kea, *Nestor notabilis;* dalmatian pelican, *Pelecanus crispus*; white-tailed eagle, *Haliaeetus albicilla*; and bald eagle, *Haliaeetus leucocephalus*) and 31 Least Concern (LC) species (Additional file 1: Table S3). We found common genomic signatures among the endangered or recently endangered species and that in the ibis was associated with feeding behavior, climate change, environmental hazard, and man-made disasters. We also found that the ibis populations are rapidly evolving and possess greater genetic diversity than expected in the recovery process. To better assist protection and recovery efforts for the crested ibis, we developed technical platforms and molecular tools, which may also be useful for the rescue and protection of other endangered wildlife.

## Results

### The reference genome assemblies and annotations of the crested ibis and its cohabitant the little egret

With the crested ibis being our focused EV species, we first assembled its reference genome *de novo* from high-coverage (approximately 156×) sequence reads generated from a series of libraries constructed with various insert sizes, using SoapDenovo [17]. The assembly reached a contig N50 size of 67 kb and a scaffold N50 size of 10.7 Mb (Table 1 and Additional file 1: Table S4), and upon adding 282 Gb single-molecule optical mapping data, its super-scaffold N50 size increased to 26 Mb. Using the conserved chromosomal organization of the chicken and zebra finch genomes, we anchored 1 Gb super-scaffolds to the chromosomes, which constitute 82% of the estimated genome size. The final reference genome represents one of the more complete assemblies in the current avian genome study [18]. We validated the reference genome with alignment to eight fully-assembled fosmid sequences (98.4% alignment; Additional file 1: Table S5) and transcriptomic data (RNA-seq from two other blood samples; 95.0% alignment; Additional file 1: Table S6). Using similar procedures, we also generated a reference genome (approximately 70× coverage) from a male little egret, which has N50 contig and scaffold sizes of 24 kb and 3.0 Mb, respectively (Additional file 1: Table S4). Our annotation efforts predicted 17,163 and 17,032 genes for the crested ibis and the little egret, respectively (Additional file 2: Figure S1). For the crested ibis, approximately 55% of the predicted genes were validated based on about 107 million mRNA sequences from blood (≥1 RPKM (reads per kilobase per million)). In addition, the repeat contents of the two birds constitute 6.6% and 7.4% of the genome length for the ibis and egret genomes, respectively (Additional file 1: Table S7), similar to that of the zebra finch [19] but less than that of the chicken [20]. The comparative genome assembly statistics and annotations of the other 41 EV and LC avian species are reported in [16]. Our study represents the first effort of comparative genomic analyses based on the genome information generated from these EV and LC species.

**Table 1:** **Data statistics of the crested ibis and the little egret**

	**Crested ibis**		**Little egret**	
*De novo* sequence				
Insert size (bp)	Total data (Gb)	Sequence coverage ( x genome)	Total data (Gb)	Sequence coverage (x genome)
170-800	141	113	53	42
2-20 × 10^3^	54	43	36	29
Total	195	156	89	71
Assembly				
	N50 (kb)	Total size (Gb)	N50 (kb)	Total size (Gb)
Contigs	67	1.24	24	1.16
Scaffolds	10,687	1.26	3,051	1.21
Annotation				
	Number	Total length (Mb)	Number	Total length (Mb)
Repeats	840,220	80	861,413	86
Genes	17,179	343	17,032	326
Coding exons	148,003	24	139,080	23
Resequencing^a^				
	Total data (Gb)	Sequence coverage (x genome)	Total data (Gb)	Sequence coverage (x genome)
Each individual	25	20	30	24
All individuals	200	160	149	119
SNP calling				
	Total SNPs	Mutation rate^b^ (θ_S_) (×10^-3^)	Total SNPs	Mutation rate^b^ (θ_S_) (×10^-3^)
Autosome	1,188,930	0.33	8,876,153	2.91
Coding exons	13,459	0.17	91,820	1.45

^a^Paired-end libraries with insert size of 500 bp were used for resequencing. Samples were from eight crested ibis and five little egrets.

^b^The population mutation parameter θ_S_ = K/aL, a = 1 + 1/2 + … + 1/(n-1), where K is the number of variant sites found by sequencing n chromosomes in a region of length L.

### Low heterozygosity among the EV species, and its ongoing loss but with signs of increased diversity in the recovered crested ibis population

**Figure 2:**
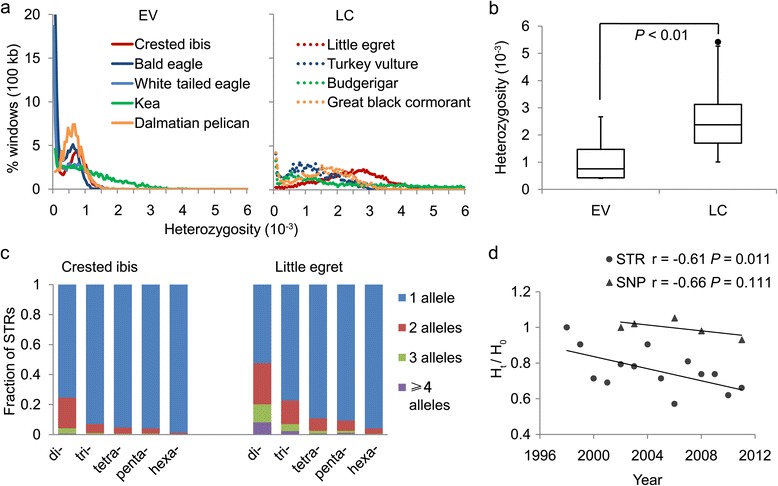
**Genomic diversity of selected EV and LC avian species. (a)** Percentage distribution of genome sequences in a 100-kb window as a function of heterozygosity (SNPs/1,000 bp) of nine representative avian species from four orders each: EVs (n = 5) and LC (n = 4) species. Species from the same order are denoted in matching colors (solid, EV; dashed, LC). Note the differences among peaks between 0 and 1 on the heterozygosity axis. **(b)** Box plot of the average heterozygosity of LC (n = 32) and EV (n = 8) species (t test, *P* <0.01). **(c)** STR-based genomic diversity. Genome-wide STR alleles are based on lobSTR software [26] from resequencing reads of the crested ibis (n = 6; randomly selected from eight samples) and the little egret (n = 6). *P* values from chi-square test for di-, tri-, tetra-, penta-, and hexa-nucleotides are all <0.001. **(d)** Gradual loss of genetic diversity (H_t_/H_0_). H_0_ and H_t_ represent initial heterozygosity and that after generation *t*. Solid circles (STR) or triangles (SNP) represent average heterozygosity of individuals from the same generation. *P* values are calculated based on linear regression.

Genetic diversity has been shown to buffer species against widespread epidemics of infectious agents and parasites; its decrease has been thought to have detrimental effects on population health and survival [21,22]. Prior studies have proposed that EV species have low genetic diversity [9,10], but all were based a limited number of neutral genetic markers. We measure genetic diversity of the EV species by aligning high-quality reads from the genome sequences of individual birds against their reference genomes [23-25]. We first compared two to three species in the same order according to the phylogeny based on whole genome sequences from a companion study [15], but with different ICUN conservation statuses. In all cases, the EV species within the pairing scheme showed significant heterozygosity reduction relative to the LC control species, with the severest found in the crested ibis and the kea (Table 2). The numbers of heterozygous SNP loci are 478,836 (heterozygosity, 0.43 × 10^-3^) in the crested ibis genome, which is approximately 6 times less than in the little egret genome (2,683,899; heterozygosity, 2.51 × 10^-3^; Figure 2a). This finding was confirmed in analyses that compared all eight EV and 32 LC species regardless of phylogenetic relationship, showing that the average heterozygosity rate of the EV species is 1.08 × 10^-3^, significantly smaller than that of the LC species of 2.49 × 10^-3^ (Figure 2b; Additional file 1: Table S8). Our analyses on heterozygous SNPs of protein-coding sequences confirmed similar heterozygosity reduction in the EV species (Table 1; Additional file 2: Figure S2).

**Table 2:** **Heterozygous SNPs in nine representative avian species**

**Common name**	**Latin name**	**Order**	**Conservation status**	**Heterozygosity of whole genome (10** ^**-3**^ **)** ^**a**^	**Heterozygosity of whole exons (10** ^**-3**^ **)** ^**a**^	**NS/S** ^**b**^
Crested ibis	*Nipponia nippon*	*Ciconiiformes*	EV (EN)	0.43	0.24	0.69
Little egret	*Egretta garzetta*	*Ciconiiformes*	LC	2.51	0.92	0.41
Dalmatian pelican	*Pelecanuscrispus*	*Pelecaniformes*	EV (VU)	0.6	0.34	0.84
Great black cormorant	*Phalacrocoraxcarbo*	*Pelecaniformes*	LC	1.39	0.56	0.57
Kea	*Nestor notabilis*	*Psittaciformes*	EV (VU)	0.91	0.45	0.63
Budgerigar	*Melopsittacusundulatus*	*Psittaciformes*	LC	4.31	2.21	0.32
Bald eagle	*Haliaeetusleucocephalus*	*Accipitriformes*	EV (Once EN)	0.43	0.22	0.84
White-tailed Eagle	*Haliaeetusalbicilla*	*Accipitriformes*	EV (Once VU)	0.4	0.21	0.80
Turkey vulture	*Cathartes aura*	*Accipitriformes*	LC	1.18	0.59	0.58

We classified endangered (EN) and vulnerable (VU) into endangered-vulnerable (EV) for the convenience of genetic analysis.

^a^ *P* <0.05.

^b^ *P* <0.01 (t test, EV versus LC).

LC, Least Concern; NS/S, the ratio of non-synonymous/synonymous heterozygosity.

To confirm this genomic signature at the population level, we analyzed SNP and STR calls, using the moderate-coverage genome sequences (approximately 20×) of eight crested ibis and five little egret individuals, which were sampled from the same populations as used for the reference genomes. We found a dramatic one-eighth (8 times less) SNP density reduction of the autosomes in the crested ibis population as compared to the little egret population (Additional file 1: Tables S9 and S10). The average frequency of short (1 to 2 bp) STR loci for the crested ibis genomes (0.7%; 2-bp, ≥4 alleles; n = 6) was an order of magnitude lower than that of the little egret genomes (8.0%, n = 6; Figure 2c). The longer STR loci (3- to 6-bp) also showed magnitudes lower frequencies, similar to what were seen in the crested ibis (Figure 2c).

Taking advantage of the extensive identity-tracking data from the living crested ibis populations, we asked if there is still ongoing heterozygosity reduction over time during species recovery. By analyzing 31 well-defined STR loci (4-bp unit), we estimated H_t_/H_0_ [4,27] (heterozygosity at generation *t*/initial heterozygosity) from 105 individuals (Additional file 1: Table S11) and found a negative H_t_/H_0_ correlation (*r* = -0.61) with population recovery time (with a slope of 0.017 units lost per year; Figure 2d). The SNP-based H_t_/H_0_ of the eight re-sequenced ibis individuals also displays a strong negative correlation with population recovery time (*r* = -0.66), although there was no significant change with time, most likely due to the limited data points (Figure 2d). To further investigate the genetic basis of this heterozygosity reduction, we calculated fixation index among four sub-populations derived from the two original breeding pairs, including their offspring kept in the original Yangxian Nature Reserve population. We found remarkably large fixation indices among the sub-populations (Additional file 2: Figure S3), despite that the first population split occurred only about 20 years ago. This points to the presence of signs for rapidly increased genomic diversity between separated populations, even though its smaller population size and physical isolation all lead to ongoing heterozygosity reduction.

### Accumulation of deleterious mutations in the threatened species

**Figure 3:**
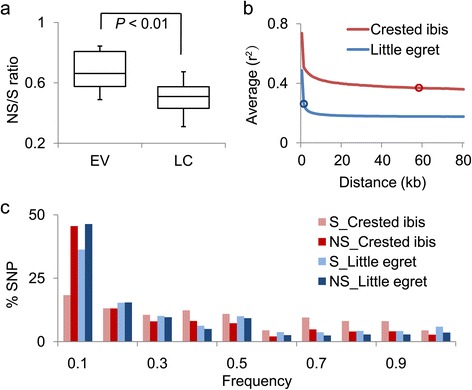
**Accumulation of deleterious mutations. (a)** Box plot of NS/S (non-synonymous/synonymous) ratio (based on heterozygous SNPs) in the LC (n = 32) and EV (n = 8) species (t test, *P* <0.01). **(b)** LD (Linkage disequilibrium) decay of the crested ibis and the little egret genomes. Open circles denote distances where the *r* ^2^ correlation coefficient reduces to half of its maximum (approximately 60 kb for the crested ibis and approximately 1 kb for the little egret). **(c)** SNP fractions as derived allele frequencies in populations of the crested ibis (n = 9) and the little egret (n = 6). NS, non-synonymous; S, synonymous.

Non-synonymous changes often lead to functional, sometimes deleterious, changes in proteins [28], and inbreeding is thought to contribute to the increase of deleterious mutations in a population [29]. We wondered if there are genes bearing more non-synonymous mutations in EV species and assessed the ratio of non-synonymous/synonymous (NS/S) heterozygous SNPs between the two haploid sequences within a diploid genome of a given species. We found that the eight EV species show much higher NS/S (0.68, median) than their closely related LC species (Table 2) or the combined ratio of all 32 LC species (0.50; Figure 3a, Additional file 1: Table S8), with the highest found in the two eagles and dalmation pelican, followed by the kea and the crested ibis (Table 2). Similarly, at the population level, the NS/S ratio of the eight other crested ibis individuals ranges from 0.66 to 0.70, as compared to 0.44 to 0.48 for the five little egret individuals, and this difference is significant (Additional file 1: Table S10).

We tested whether the higher NS/S ratios can be attributed to stronger linkage disequilibrium due to inbreeding in a small effective population [30], using the multiple sequenced individuals. The crested ibis population has a slow linkage disequilibrium (LD) decay with reduced *r*^2^ correlation coefficient at half of its maximum and at a distance of approximately 60 kb as compared to the little egret population with a distance of approximately 1 kb (Figure 3b). A similar slow LD decay has been observed in highly-inbred domestic species, such as horse [31] and dog [32]. Furthermore, the synonymous SNP fraction of the derived alleles at a low frequency of 0.1 in the crested ibis population is just half that of the non-synonymous SNPs, whereas the two values are either higher or comparable in the little egret (Figure 3c). It appears that the decreased proportion of low-frequency synonymous SNPs relative to non-synonymous SNPs is a result of inbreeding fixation in the small crested ibis population.

### Genes involved in brain function and cytochrome P450 metabolism have allelic fixation in the recovered crested ibis population

**Figure 4:**
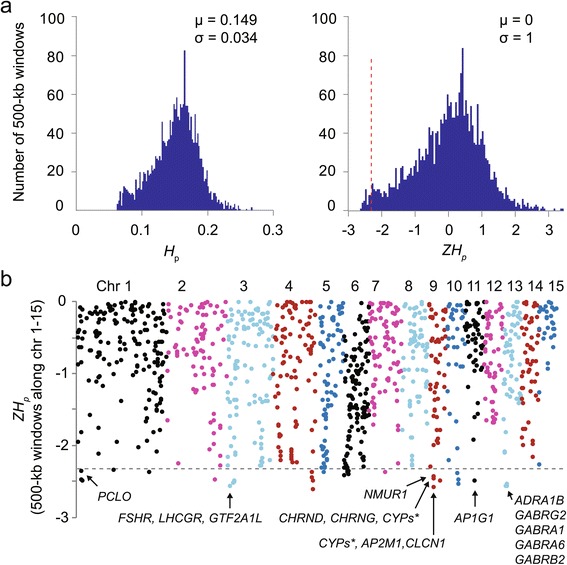
**Heterozygosity loss and selected genes in the crested ibis genome. (a)** Distributions of heterozygosity, *H*_p_ (left), and corresponding Z transformations, *ZH*_*p*_ (right), for all 500-kb windows (n = 2,513). μ, mean; σ, standard deviation; red vertical dashed line, threshold at *ZH*_*p*_ = -2.326 (q <0.01 in normal distribution). **(b)** The negative end (error head in a) of the *ZH*_*p*_ distribution presented along chromosomes 1-15 (color-coded from left to right). The horizontal dashed line indicates the threshold (see a). Genes residing within a window with *ZH*_*p*_ < -2.326 are indicated (Additional file 1: Table S14).

To find out whether the SNP fixation we observed in the recovered ibis population was randomly distributed among the genomes or specific to certain segments and genes, we utilized a method that identifies differences in rates of fixed SNPs, which are assumed, but does not necessarily have to occur by selective sweeps within the genomes [33]. Specifically, we calculated heterozygosity (*H*_*p*_) and its Z transformations, *ZH*_*p*_ (Figure 4, see Materials and methods) in 500-kb sliding windows (n = 2,514) along whole genomes (except for sex-chromosome scaffolds) for the most and least frequently observed alleles at all SNP positions. From the distribution of observed *H*_*p*_ and *ZH*_*p*_ (Figure 4a), we defined a threshold of fixed SNPs (*ZH*_*p*_ score equal to -2.326 or less, *P* <0.01, Figure 4b). The smallest *H*_*p*_ values represent the least frequently observed alleles. We found approximately 1.4% of the windows (n = 36) had a *ZH*_*p*_ score -2.326 or less (Figure 4b), and thus were significant outliers for the whole genome. We examined all candidate genes that resided in these regions (Additional file 1: Tables S12), and categorized them according to Gene Ontology (GO) terms (Additional file 1: Tables S13 and S14). Seven categories were statistically enriched, and nearly all involved in brain function: postsynaptic membrane, synapse part, GABA-A receptor activity, GABA signaling pathway, extracellular ligand-gated ion channel activity, chloride transport, and clathrin adaptor complex. Of the genes involved, 10 were over-represented in the neuroactive ligand-receptor interaction pathway (KEGG map04080) that processes information from exogenous signals using neurotransmitters and signaling molecules, including epinephrine, norepinephrine, neuromedin, melanin-concentrating hormone, GABA, and acethycholine (Additional file 1: Table S13). In addition, four of the cytochrome P450 genes stood out, which function in metabolism of hormones and toxins, including arachidonic acid (map00590) and linoleic acid (map00591) metabolism pathways that process essential fatty acids and play an important role in immune modulation [34]. These findings suggest that the fixation in the genome is not random.

### Population bottlenecks of the crested ibis and immune genes

**Figure 5:**
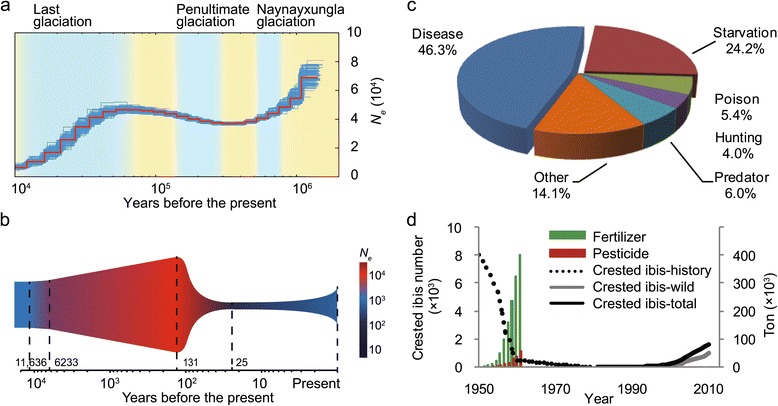
**Demographic history reconstruction of the Chinese crested ibis population based on the resequenced data from eight resequenced individuals. (a)** Estimation based on the PSMC (pairwise sequentially Markov coalescent) model. The red line depicts the estimated effective population size (*N*_*e*_), and the thin blue curves represent PSMC bootstrapping estimates. The sky blue and yellow background colors indicate glacial and interglacial periods, respectively. **(b)** Estimation based on the ∂a∂i calculator. The timing of demographic events is indicated (vertical dashed lines; x-axis indicates time in logarithmic scale). **(c)** Percent of deaths from various causes of the wild crested ibis from 1981 to 2003 [14]. **(d)** Agrochemical usage and population size. The population size was negatively correlated with the usage of pesticides and fertilizers during 1950s to early 1960s in China (fertilizer, r = -0.92, *P* <0.001; pesticide, r = -0.95, *P* <0.001). Agrochemical use has been forbidden in the sanctuary designated for the recued ibis population since 1981. *P* values were calculated based on linear regression (data on pesticide and fertilizer usage are summarized in Additional file 1: Table S17).

It is possible that the alleles became fixed through a bottleneck affect reducing the polymorphisms in the genome driven by genetic drift. Population bottleneck refers to a sharp population size reduction due to environmental events or human activities. With sequence data from whole genomes, and of multiple individuals, we can calculate population bottlenecks more reliably than with several genes and neutral markers. We reconstructed the crested ibis’ demographic history using our sequence data based on a pairwise sequential Markovian coalescent (PSMC) model [35] and a diffusion approximation method for demographic inference (*∂a∂i*) [36] (see Materials and methods). This analysis revealed two ancient and one recent bottleneck (Figure 5a). The two ancient events (1 to 0.01 MYA) occurred during the Naynayxungla glaciation (0.78 to 0.50 MYA) [37] and the last glaciation (Ice Age, 0.01 MYA), resulting in a precipitous population drop to approximately 10,000 individuals at the end of the last glaciation. The recent event leads to a drastic population reduction throughout the last 100 years, terminating approximately 25 years ago with the human assisted recovery effort (Figure 5b, Additional file 1: Table S15). This timeframe is not only consistent with the known historic records of the ibis species [2], but also reflects a blend of global climate change [37], intensified human activity [3], and remarkably, population recovery after conservation efforts commenced some 30 years ago. This result is very different from the little egret in which population decreased during the Penultimate glaciation (0.3 to 0.13 MYA), but only slightly decreased in the last glaciation (Additional file 2: Figure S4).

To search for possible genetic causes for the recorded animal deaths among the recovered population, we scrutinized the records and found that the crested ibis population in the wild, while in the human-assisted recovery, has still been suffering from parasitic infection and other diseases, which account for 46.3% of total deaths from 1981 to 2003 (Figure 5c) [13]. Since the major histocompatibility complexes (MHC) and its genetic variants are critical for immunity [22], we analyzed the nucleotide sequences of the classical MHC class II β gene (*BLB*) antigen binding region (encoding a protein for presenting antigenic peptides to helper T cells). This gene shows a much lower genetic diversity (1 locus with ≥3 amino acid alleles) in the antigen binding domain than its homolog of the little egret (6 loci with ≥3 amino acid alleles; Additional file 2: Figure S5). Such a low level of genetic diversity in the *BLB* and other MHC genes may result in defective immunity of the crested ibis population.

### Historic agrochemical overuse and relevant mutated genes in threatened avian species

Overuse of various nondegradable agrochemicals has been suggested as one of the principal reasons for the population decline of seven of the eight EV species examined: bald eagle [38], white-tailed eagle [39], kea [40], Adele penguin [41], emperor penguin [42], chimney swift [43], and the Dalmatian pelican [44]. To investigate whether agrochemical use also contributed to the decline of the crested ibis survival, we first examined the relevant historical evidence. In Japan, during the Meiji Restoration (in the late 19th century), traditional protection measures were disregarded and rampant hunting rapidly reduced the crested ibis population to the extent that by the time when the species was enlisted for protection on the hunting ordinances in 1908, it was almost extinct there [2]. Although relevant evidence was poorly documented in Korea, Northern China, and Russia in the first half of the 20th century, in central China, the crested ibis was common in Gansu and Shaanxi Provinces before 1950 but nearly extinct by the end of 1950s. We found a negative correlation between the estimated crested ibis population size in the Gansu and Shaanxi Provinces and the amount of fertilizers and pesticides used in the region (Figure 5d). These findings suggest that overuse of agrochemicals may be associated with very dramatic and obvious decline in the crested ibis population of the region from which our genomes were sequenced.

We compared 6,332 orthologs genes among EV (all are carnivorous species; n = 8) and also to the LC carnivorous species (n = 15), since carnivorous species are also often apex predators and more sensitive to agrochemicals [38,39,41,44]. We identified 44 genes that have a significantly higher rate of being inactivated (null mutations that alter protein structure) in the EV carnivorous species, and only nine genes with a significantly higher rate in the LC carnivorous species (Fisher’s exact test, *P* <0.05; Additional file 1: Table S16). Among them, 17 genes are metabolism related enzymes; for instance, one of them, *SLCO1A2*, a sodium-independent transporter mediating cellular uptake of organic anions in the liver [45], has lost its function in three threatened species (37.5%) and in none (0%) of the LC species. Another, *HACL1*, catalyzing a carbon-carbon cleavage reaction, is necessary for the catabolism of phytanoic acid in carnivores [46], which has lost its function in three threatened species. *CHIA*, which degrades chitin-chitotriose and participates in the defense against pathogens [47], has lost its function in five threatened species. These findings suggest that carnivorous EV species have a greater genetic susceptibility to agrochemicals.

### Genome-wide STR profiling of the crested ibis population for marker-assisted breeding

**Figure 6:**
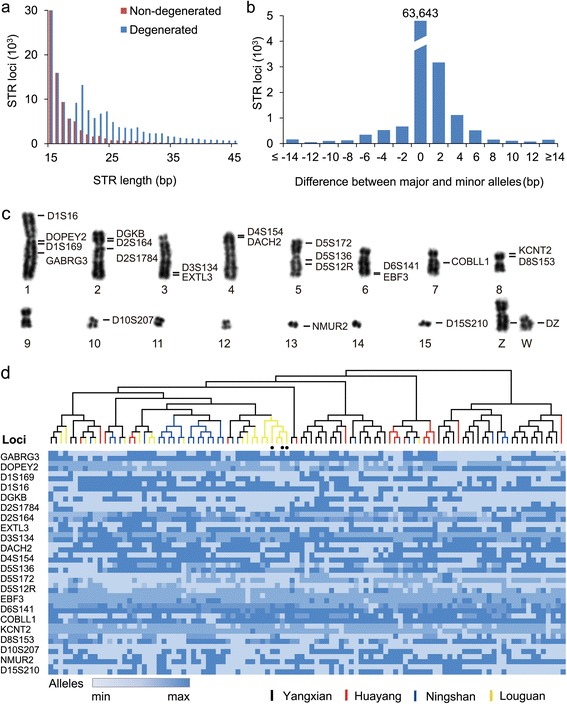
**Genome-wide STR profiling of four ibis sub-populations. (a)** STR (units of 2 bp, 3 bp, 4 bp, 5 bp, and 6 bp) distribution as a fraction of the total repeat length. Non-degenerate STRs do not contain insertions, deletions or mismatches. **(b)** Near random distribution of allele size differences between the major and minor alleles (n = 9). Size difference is calculated by subtracting the minor allele length from the major allele length. **(c)** Genetic markers of the ibis chromosomes typed in this study. Twenty-two representative STR and a single sex chromosome (W)-derived markers are shown here. **(d)** Individual identification based on the 22 STR loci. The colored horizontal scale bar indicates the number of repeat units (from minimum to maximum). The alleles (105 individuals) are used to construct neighbor-joining tree within sub-populations (Yangxian, n = 42; Ningshan, n = 27; Huayang, n = 16; and Louguan, n = 20). Solid circles denote the three individuals from a single family.

Conservation and rescue of a species from near-extinction has been partly assisted with having genetic loci to track individuals to reduce inbreeding [11]. However, there have been limited number of markers that can do so, including for the crested ibis. Further, changing conservation practice from small-scale captive breeding to STR-assisted breeding in large Nature Reserves can theoretically be benefitted by genome-scale approaches for genetic markers. In this study, we identified approximately 166,000 degenerate STR loci (sequences containing insertions, deletions, or mismatches) from the crested ibis genome sequences (Figure 6a) and tested them against a population of nine crested ibis individuals (18 haplotypes). On average, minor STR alleles exhibited a 4-bp difference from their major alleles and 18% of the allelic differences differed by >5 bp over their major alleles (Figure 6b). Focusing on the 4-bp STRs, we confirmed heterozygosity for 300 such loci within and across some of the crested ibis sub-populations (105 individuals) and selected a set of 23 informative STR loci, including 22 autosomal loci and one sex-linked locus (distinguishing male from female with a 31-bp insertion in W chromosome) to establish a DNA identification profiling (DIP) platform (Figure 6c and d, and Additional file 2: Figure S6; Additional file 1: Table S18). The new sex marker accurately discriminated between males (ZZ) and females (ZW) (Additional file 2: Figure S7), an important advance since morphological-based gender determination for the crested ibis is quite difficult. This DIP marker set had a collective discrimination power of 0.628 and an exclusion probability of 0.994 (Additional file 1: Table S19). This platform demonstrated an estimated probability of paternity up to 99.997% on pedigree analysis in a four-generation family (Additional file 1: Table S20). Our DIP platform is now being used in reconstructing pedigree relationships, establishing individual identity for the recovering crested ibis populations and assisting non-sibling or genetically distant parental pairing.

## Discussion

Our genome-wide analysis on the endangered crested ibis and seven other recently endangered and rescued avian species across the Neoaves phylogenetic tree provides direct evidence at a genomic-scale for support of previous hypothesis and novel insights into consequences of heterozygosity loss, deleterious mutation accumulations, population bottlenecks, and genetic drifts. The convergent inactivation (or pseudogenization) of xenobiotic metabolism related genes in the ibis and other endangered top predators suggests a reduction of adaptive genetic plasticity in these species to agrochemical overuse. However, the increasing genomic diversity among the isolated ibis populations derived from the offspring of the last wild pair identified in 1981 indicate that rapidly diverging sequences in the recovering ibis population are being fixed in less than 10 generations.

Our genome-wide data are important for exploring causative factors of the near-extinction and exact demographic reconstruction of the endangered species, and both are necessary for distinguishing long-term climate change from recent human-mediated events [11,48]. In our case, we identified distant bottlenecks due to the past glaciations and the most recent bottleneck that is clearly unrelated to global glaciations (although severe periodic temperature drops may happen to accelerate the process) but associated with some man-made factors. The man-made induced bottleneck was much more severe than the glaciations. It appears that the crested ibis is more sensitive to these environmental challenges than the little egret. Similar events may have affected endangered non-avian species, such as the giant panda [49]. However, our observations on the EV avian species provide an avian model for conservation genomics, which is distinctly different from giant panda whose genetic diversity remains high [25,49] albeit with a similar population size (approximately 2,000 for the crested ibis vs. approximately 2,500 for the giant panda). We propose that, regardless of the past conservation success, an immediate evaluation of genetic diversity and sequence variation should be imposed for risk assessment on all endangered species.

The genetic drift for fixation of changes in brain and metabolism genes of the rescued crested ibis population in China is intriguing, and could mean either deleterious mutations in these genes or the possibility of enhanced functions for certain brain behaviors and enhanced metabolism of toxins for survival of the species. Consistent with the former possibility, the surviving crested ibis in China may have gone through a change in foraging behavior [2]. As a wading bird, the crested ibis uses a ‘remote touch’ mechanism to detect the movement of their prey in the mud through a series of sensory receptors [50], and either sacrifice or gain of sensing and digesting abilities are all relevant. We do not know if this change occurred demographically before or through genetic drift after the two breeding pairs were rescued in 1981.

One interpretation of the fixation findings based on the methods we used is that there have been selective sweeps for specific SNPs in specific brain and metabolism genes over the past 30 years. These sweeps could have artificially occurred due to controlled inbreeding, or naturally occur due to selection. But such selective sweeps within such a short time, for animals that reach sexual maturity at around 3 years with limited generations seems remarkable; although we see more rapid increasing diversity in the genome than expected. An alternative, more likely interpretation is that greater fixation of these alleles was already present in the two last breeding pairs before near extinction due to demographic differences [51]. This difference is difficult to test considering all the animals we sequenced are descended from the last seven individuals in the wild from one population in 1981, where all others are extinct. If a demographic explanation were true, it would mean that these alleles became fixed through a bottleneck affect reducing the polymorphisms in the genomes by genetic drift.

One question that can now be better addressed is why the crested ibis nearly suffered extinction, whereas its cohabitant, the little egret, did not? One possible reason is that while both species exploit aquatic environments, such as eating mollusks, crustaceans, fish, and frogs, the little egret also consumes plant seeds in the winter or under drought and thus still thrives strongly in the same habitat. This foraging behavioral difference is consistent with genetic differences in enzymes for food digestion. Another possible reason is that the little egret might have become resident birds and gone through a bottleneck already by changing their foraging behavior at the same time.

Our genome-wide STR markers and its application to conservation genomics also provide a more powerful platform for breeding and tracking of endangered species both in partial captivity and in the wild. With this method, we believe that it is possible that immediate genome sequencing and evaluation of genetic diversity and loss-of-function genes for risk assessment can be done for generating rescuing strategies for other currently endangered avian species.

## Conclusions

Our study is the first, of which we are aware, to conduct genome-scale analyses of species that were endangered, including near extinct, across a vertebrate class. With a focus on the crested ibis, we were able to identify genetic associations before, during, and after the near extinction events and population bottlenecks. We confirmed some expected changes, but genome-wide, such as reduced heterozygosity, accumulation of deleterious mutations, and susceptibility to agrochemical overuse by humans. We also identified seemingly positive changes in the recovering crested ibis population, such as more rapidly increasing genetic diversity between new populations than expected, and changes in some gene families that could potentially be related to surviving extinction or recovery. Our genome-scale derived STR platform is now assisting in that recovery. We hope that the knowledge and lessons learned from this study will be applicable to not only the one-quarter of avian species that are threatened or near threatened, but to threatened species broadly.

## Materials and methods

### Sample collection

For *de novo* assembly, we extracted DNA samples from peripheral venous blood of a 3-year-old female crested ibis in the Yangxian County Reserve and a male little egret captured from the same county in southern Qinling Mountains, Shaanxi Province, China. For our resequencing effort, blood DNA samples were from eight crested ibis and five little egret individuals from the same locality. For meta-analysis of endangered and least concerned species, we used the genome sequences of 41 additional avian species (Additional file 1: Table S3) [16]. For DNA profiling, we used 105 individual crested ibis from four sub-populations of Yangxian, Huayang, Louguan, and Ningshan Counties (Additional file 1: Table S11).

### Karyotyping

Skin cells were grown in DMEM medium supplemented with 15% fetal bovine serum. Metaphase preparations for flow sorting were generated [52]. The crested ibis chromosomes were numbered according to convention [53]. Chromosome preparations were stained with Hoechst 33258 (Sigma, St Louis, MO, USA) and Chromomycin A3 (Sigma) and then sorted [54] (MoFlo, DAKO, Glostrup, Denmark DAKO).

### Genome sequencing

For genome assembly, we constructed sequencing libraries with variable insert sizes (180 bp, 500 bp, 800 bp, 2 kb, 5 kb, 10 kb, and 20 kb) by following the manufacturer’s instruction for Illumina’s HiSeq 2000. Sequences of approximately 266 Gb and 127 Gb (reads length: 100 bp for libraries with insert size <2 kb, 45 bp for other libraries) were generated for the crested ibis and the little egret, respectively, and after quality-filtering, approximately 194 Gb (roughly 156×) and about 90 Gb (71×) sequences survived for the assembly. To achieve better contiguity, we also used a new physical mapping technology developed by the Argus System (OpGen) and its assembly software (Genome-Builder), which produced, based on KpnI digest, a large optical mapping dataset (about 282 Gb) from 34 high-density MapCards and containing 799,678 single-molecule restriction maps (>250 kb) with an average size of 353 kb.

### Genome assembly

The genome sequences for the crested ibis and little egret were assembled by using the de Bruijn graph-based assembler SOAPdenovo [17]. Prior to assembly, potential sequencing errors were removed or corrected based on k-mer frequency methodology. Reads from libraries with insert sizes ranging from 170 bp to 800 bp were split into 41-mers to construct de Bruijn graphs and contigs. The paired-end reads were aligned to construct scaffolds. Super-scaffolds for the crested ibis were constructed and aided with optical mapping data. The crested ibis chromosomes were built by using super-scaffolds based on conserved synteny between the assembly and genome data of chicken and zebra finch.

To assess the large-scale and local assembly accuracy, we also sequenced (Sanger sequencing technology) and assembled (phred-phrap-consed) eight randomly selected fosmids (average approximately 39 kb long) from a genomic library for the crested ibis (same DNA used for the reference assembly). We also assessed the completeness and accuracy of our assembly using 98,881 transcripts from blood, which were sequenced and assembled independently. A total of 94,709 assembled transcripts (>95%) were mapped to the assembly (BLASTN, E <10^-5^, coverage ≥90%), yielding a single-base accuracy of approximately 98% for the assembled sequences with >20 reads coverage and excluding sequence gaps.

### Gene and repeat annotations

To predict genes, we used both homology-based and *de novo* methods as follows. First, we obtained protein sequences of chicken, zebra finch, and human from Ensembl (release 60) and mapped them onto the genome assemblies using Tblastn with E-value 1e-5. All high-score segments were grouped into gene-like structures (genBlastA [55]). The homologous genome sequences with flanking sequences (2,000 bp) were aligned to the protein sequences by using Genewise [56] to define gene models. We clustered predicted transcripts >100 bp and took cross-species synteny into account (otherwise, a transcript with the best aligning score was used). Single-exon genes containing one or >1 frame shift errors and multi-exon genes containing >3 frame errors were not taken into account. Second, we clustered transcripts using TopHat [57] and Cufflinks [58] and aligned them (>150 bp) to SwissProt/TrEMBL database [59] with a cutoff E-value <1e-5. Third, we predicted protein-coding genes (>150 bp) using Genscan [60] (gene model parameters trained with *Homo sapiens* genes) and Augustus [61] (gene model parameters trained with chicken genes) and defined TE-derived proteins (BlastP with E-value <1e-5 and >50% alignment).

For the reference gene set, we constructed gene models following three criteria: (1) candidate genes clustered with >100 bp overlap; (2) one cluster per gene (homology-based model > RNA-seq model > *de novo* predicted model); and (3) if not (2), 30% alignment to a known protein in the SwissProt/TrEMBL database [59] (>2 exons). Functional annotations were based on the best match principle using SwissProt, InterPro [62], and KEGG [63] databases. Treefam [64] was used to define gene family (Blastp, E-value <1e-7; Hscore >10; minimum edge density >1/3) and CAFE [65] to define gene loss and gain.

We annotated transposable elements (TEs) based on homology to RepBase sequencesusing RepeatProteinMask and RepeatMasker [66] with default parameters. We also constructed *de novo* repeat libraries (transposable elements) using RepeatModeler (http://repeatmasker.org/RepeatModeler.html) with default parameters.

### Resequencing data analysis

Resequencing reads were generated from a single-size insert library (500 bp) per individual and mapped high-quality reads onto the references with BWA [36], followed by removal of unmapped reads (average quality <10 or average map quality <20 or multiple-site reads). SNPs were called by using SOAPsnp [67] with thresholds of quality score ≥20, sequencing depth >8X and <40X, copy number of flanking sequences < = 2, >3 uniquely mapped reads, and distance between SNPs ≥5 bp.

We calculated the correlation coefficient (*r*^*2*^) of alleles at SNP locus after setting -maxdistance 300 -dprime -minGeno 0.6 -minMAF 0.1 -hwcutoff 0.001 using the Haploview software [68]. Since sample size is an important parameter influencing LD patterns, we randomly selected five crested ibises three times to repeat the experiment and the analysis. To reconstruct ancient demographic history, we ran the PSMC program (parameters: -N30, -t15, -r5, and -p ‘4 + 25*2 + 4 + 6’) using autosomal sequences (scaffold length ≥50 kb and a total of 478,758 heterozygous loci). We performed bootstrapping (100 times) to estimate the variance of simulated results and estimated the neutral mutation rate μ (mutations per base per generation) using the estimated genome-wide nucleotide divergence (10.31%) and divergence time (38.98 × 10^6^) between the crested ibis and the little egret. Based on mean generation time (3 years for crested ibis), we calculated μ = (0.1031 × 3)/(2 × 38.98 × 10^6^) = 3.968 × 10^-9^ mutations per generation for the crested ibis.

To reconstruct recent demographic history, we used the ∂a∂i program [36] and paired-end reads (500 bp in size) from nine samples (eight re-sequencing individuals and one *de novo* assembly individual). To minimize the effect of low-coverage sequencing, we extracted the sites that were covered by high-quality reads (at least six of nine individuals covered by >2X reads). To prepare for ∂a∂i program, we called 1,420,399 SNPs using a published method [69]. The little egret reference genome sequence was used to infer ancestral alleles. We considered four models and chose the one with highest maximum log-likelihood value. The ancestral population size (Na) was estimated on the basis of the calculated θ value and the mutation rate. Population size and corresponding time were derived from parameters scaled based on Na.

### Purifying selection analysis

For each 500-kb window, we determined the number of reads corresponding to the most and least abundant SNP alleles (n_MAJ_ and n_MIN_), *H*_p_ = 2∑n_MAJ_∑n_MIN_/(∑n_MAJ_ + ∑n_MIN_)^2^, and transformed *H*_p_ into Z scores: *ZH*_*p*_ = (*H*_p_-μ*H*_p_)/σ*H*_p_ [33]. We applied a threshold of *ZH*_*p*_ = -2.326 (q <0.01 in normal distribution) for putative selective sweeps.

### Genome-wide STR profiling

We defined STRs using Tandem Repeat Finder [70] (parameters: Match = 2, Mismatch = 7, Delta = 7, PM = 80, PI = 10, Minscore = 30, and MaxPeriod = 6), which were validated in the following steps. DNA was extracted with the E.Z.N.A.™ Blood DNA Kit (Omega Bio-Tek Inc., USA) according to its instruction (E.Z.N.A.™ Blood DNA Isolation Protocols, Revised June 2009). All DNA samples were quantified with the TIANamp Genomic DNA Kit. PCR amplification was performed in a reaction volume of 25 μL with MicroAmp® reaction tubes (Applied Biosystems, CA, USA; the GeneAmp® PCR Systems 9700 with gold-plated silver or silver 96-well blocks). Amplified products were separated in ABI3730 DNA Genetic Analyzer 48-capillary array system (Applied Biosystems) according to the manufacturer’s protocol. The genotypes were analyzed by using Genemapper 3.5 (Applied Biosystems).

### Data availability

Genome data of crested ibis and little egret are uploaded to NCBI (PRJNA232572 and PRJNA232959). The raw reads in the SRA (SRP035852 and SRP035853). The NCBI accession numbers of the assembled genomes of all species are described in Additional file 1: Table S3.

## Additional files

Additional file 1: Table S1. The important events for crested ibis. **Table S2.** Other evidence to estimate the number of crested ibis. **Table S3.** Summary of avian species and genome accessions. **Table S4.** More detailed summary of genome assemblies of the reference crested ibis and little egret genomes. **Table S5.** Fosmid sequencing and assembly. **Table S6.** Summary of transcriptome sequencing. **Table S7.** Transposable elements (TE) in the genomes of the crested ibis and the little egret. **Table S8.** Heterozygous SNPs in 40 avian species. **Table S9.** Resequencing summary of the eight crested ibis and the five little egret individual animals. **Table S10.** SNPs of the crested ibis and the little egret. **Table S11.** Sample information of 105 crested ibis individuals. **Table S12.** Details of genes in the windows of low *ZH*_*p*_ score in crested ibis genome. **Table S13.** Enrichment GO terms of genes in the windows of low *ZH*_*p*_ score in crested ibis genome.**Table S14.** Details of genes in the windows of low *ZH*_*p*_ score in crested ibis genome. **Table S15.** Results from model variations based on the ∂a∂i calculations. **Table S16.** Pseudogenes in threatened and near threatened avian carnivorous species. **Table S17.** Pesticide and fertilizer usage number in China (Shaanxi and Gansu province). **Table S18.** STR summary of DIP (crested ibis DNA identification profiling). **Table S19.** Population parameters of STR loci used in DIP. **Table S20.** Paternity test of a crested ibis pedigree. Additional file 2: Figure S1. Circular display of genomic information from the crested ibis. **Figure S2.** Box plot of the average heterozygosity in protein coding region of the genomes between LC (n = 32) and EV (n = 8) species (t test *P* = 0.005). **Figure S3. (a)** Sampling sites. **(b)** Pairwise *Fst* among four sub-populations. *Fst*, fixation index, range 0-1. A value of 0 implies that the two populations are interbreeding freely, whereas a value of 1 implies that the two populations do not share any genetic diversity. **Figure S4.** Demographic history. Estimation of the little egret based on the PSMC (pairwise sequentially Markov coalescent) model. The red line depicts the estimated effective population size (*N*_*e*_), and the thin gray curves represent PSMC bootstrapping estimates. The sky blue and yellow background colors indicate glacial and interglacial periods, respectively. **Figure S5.** Genetic variation in the peptide binding region (red rectangle) of MHC class II β gene (*BLB*) of crested ibis (upper) and little egret (bottom). The consensus sequences of nine individuals are given at the top of each alignment. There are 12 loci and 18 loci maintaining >= 2 amino acid alleles for the nine crested ibis and the nine little egrets, respectively. There are one loci and six loci maintaining >= 3 amino acid alleles for the nine crested ibis and the nine little egrets, respectively. **Figure S6.** An example of STR genotyping. The PCR amplified products were separated by the ABI3730 Genetic Analyzer. Lanes 1 and 47 represent size markers and the remaining lanes depict PCR products of individual samples. Loci were labeled in multiple colors. **Figure S7.** Sex discriminating locus of crested ibis. Electrophoresis is in the top panel. The sequence of the sex marker is in the bottom panel. The green bases represent primer sequences. The indels in Z and W chromosomes were marked in red.

## Abbreviations

*BLB*: MHC class II β gene
*CHIA*: Chitinase, acidic
DIP: DNA identification profiling (DIP) platform
EN: Endangered
EV: Combined EN and VU
GABA: γ-aminobutyric acid
GO: Gene ontology
*HACL1*: 2-hydroxyacyl-CoA lyase 1
*Hp*: Heterozygosity in 500-kb sliding windows
H_t_/H_0_: Heterozygosity at generation t/initial heterozygosity
IUCN: International Union for Conservation of Nature
KEGG: Kyoto Encyclopedia of Genes and Genomes
LC: Least concern
LD: Linkage disequilibrium
MHC: Major histocompatibility complexes
MYA: Million years ago
NS/S: Nonsynonymous/synonymous
PSMC: Pairwise sequential Markovian coalescent
RPKM: Reads per kilobase per million
*SLCO1A2*: Solute carrier organic anion transporter family member 1 A2
SNP: Single nucleotide polymorphisms
STR: Short-tandem repeat
VU: Vulnerable
*ZHp*: Z transformations of *Hp*
