# Supplementary material for: Genomic signatures of near-extinction and rebirth of the crested ibis and other endangered bird species
Source: Genome Biol. 2014 Dec 11;15(12):557. doi: 10.1186/s13059-014-0557-1 (PMC4290368; doi:10.1186/s13059-014-0557-1)
Supplement: Additional file 2: Figure S1. — Circular display of genomic information from the crested ibis. Figure S2. Box plot of the average heterozygosity in protein coding region of the genomes between LC (n = 32) and EV (n = 8) species (t test P = 0.005). Figure S3. (a) Sampling sites. (b) Pairwise Fst among four sub-populations. Fst, fixation index, range 0-1. A value of 0 implies that the two populations are interbreeding freely, whereas a value of 1 implies that the two populations do not share any genetic diversity. Figure S4. Demographic history. Estimation of the little egret based on the PSMC (pairwise sequentially Markov coalescent) model. The red line depicts the estimated effective population size (N e), and the thin gray curves represent PSMC bootstrapping estimates. The sky blue and yellow background colors indicate glacial and interglacial periods, respectively. Figure S5. Genetic variation in the peptide binding region (red rectangle) of MHC class II β gene (BLB) of crested ibis (upper) and little egret (bottom). The consensus sequences of nine individuals are given at the top of each alignment. There are 12 loci and 18 loci maintaining >= 2 amino acid alleles for the nine crested ibis and the nine little egrets, respectively. There are one loci and six loci maintaining >= 3 amino acid alleles for the nine crested ibis and the nine little egrets, respectively. Figure S6. An example of STR genotyping. The PCR amplified products were separated by the ABI3730 Genetic Analyzer. Lanes 1 and 47 represent size markers and the remaining lanes depict PCR products of individual samples. Loci were labeled in multiple colors. Figure S7. Sex discriminating locus of crested ibis. Electrophoresis is in the top panel. The sequence of the sex marker is in the bottom panel. The green bases represent primer sequences. The indels in Z and W chromosomes were marked in red. [file 13059_2014_557_MOESM2_ESM.pdf]

Additional file 2 Supplementary figures for "Genomic signatures of near-extinction and rebirth in the Crested Ibis and other endangered bird species"

Figure S1

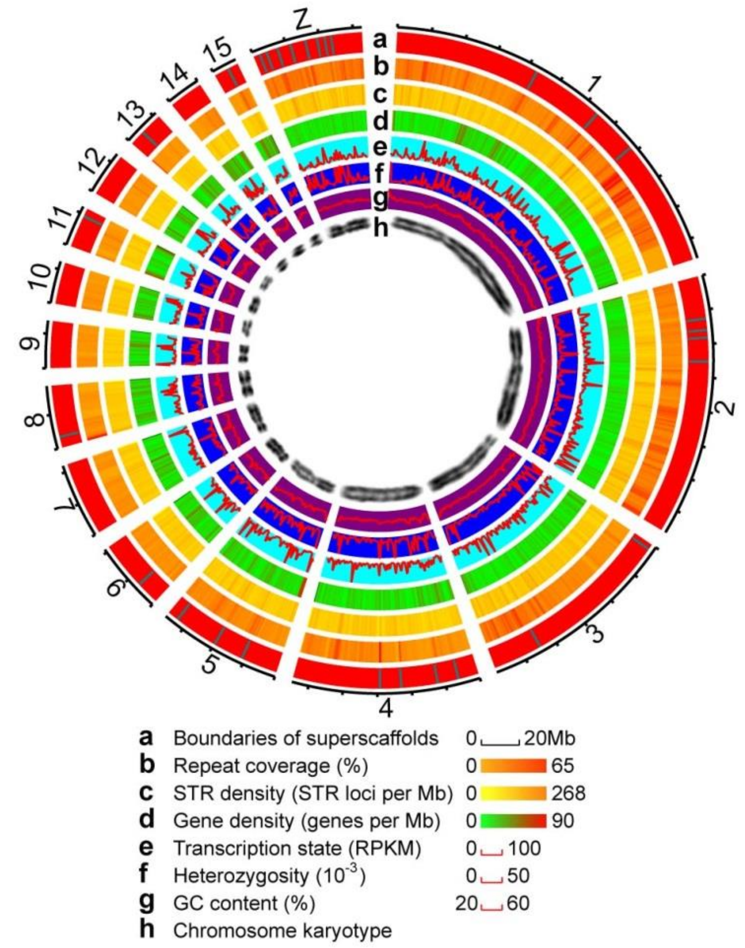

Figure S1 Circular display of genomic information from the crested ibis. From outside to inside: (**a**) physical distance and super-scaffold boundaries of the larger (1-10) and Z chromosomes (total 36 chromosomes; 26 smaller chromosomes are not displayed), (**b**) repeat coverage (% of total genome, in 1-Mb window; see methods), (**c**) STR density (STRs per Mb), (**d**) gene density (genes per Mb), (**e**) transcription status (average reads per kilobase per million reads or RPKM in 1-Mb window), (**f**) heterozygosity, heterozygous SNPs/1000bp in 1-Mb window, (**g**) genomic GC content (%G+C in 1-Mb window), and (**h**) karyotype of the larger (1-10) and Z chromosomes.

Figure S2

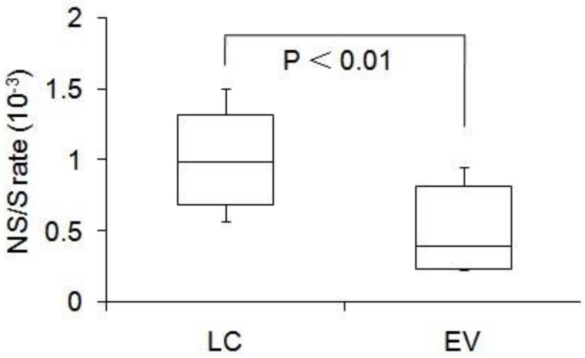

Figure S2 Box plot of the average heterozygosity in protein coding region of the genomes between LC (n = 32) and EV (n = 8) species (t test P = 0.005).

Figure S3

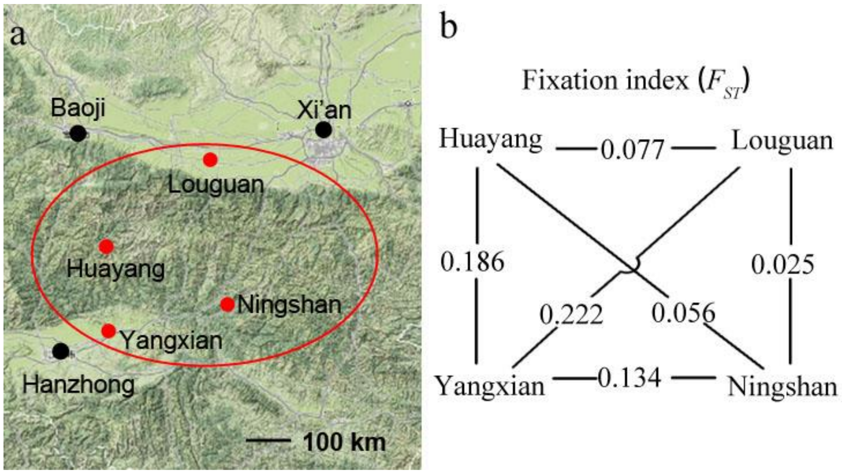

Figure S3 (**a**) Sampling sites. (**b**) Pairwise *Fst* among four sub-populations. *Fst*, fixation index, range 0-1. A value of 0 implies that the two populations are interbreeding freely, whereas a value of 1 implies that the two populations do not share any genetic diversity.

Figure S4

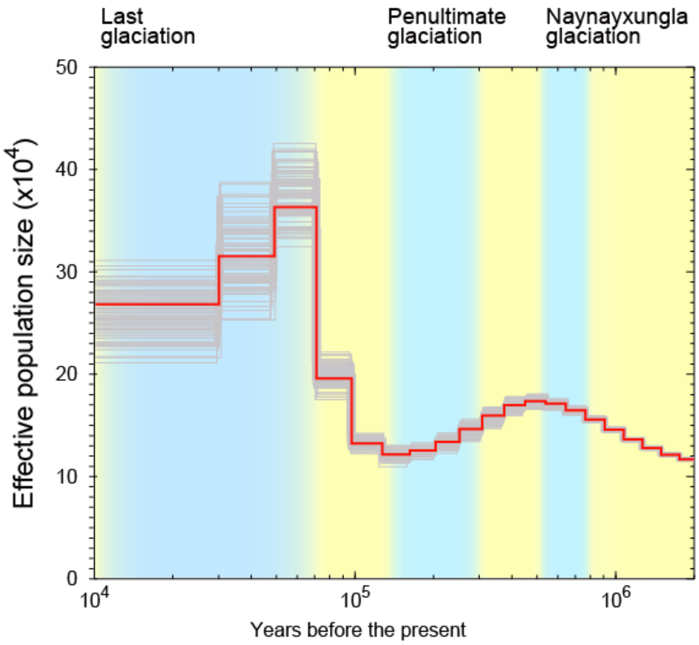

Figure S4 Demographic history Estimation of the little egret based on the PSMC (pairwise sequentially Markov coalescent) model. The red line depicts the estimated effective population size (N_e), and the thin gray curves represent PSMC bootstrapping estimates. The sky blue and yellow background colors indicate glacial and interglacial periods, respectively.

Figure S5

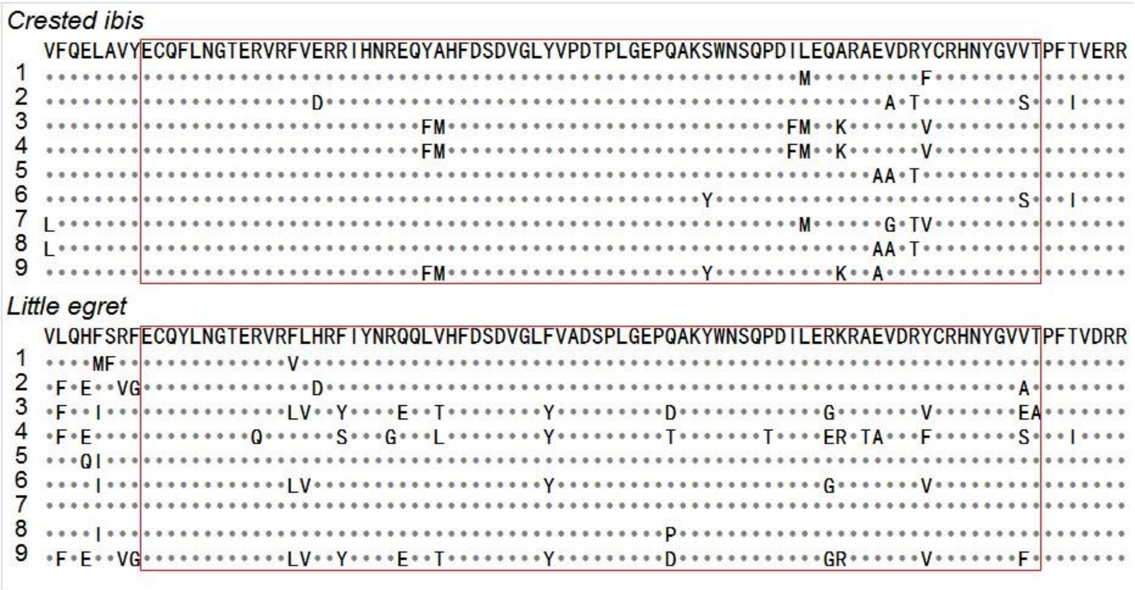

Figure S5 Genetic variation in the peptide binding region (red rectangle) of MHC class II β gene (*BLB*) of crested ibis (upper) and little egret (bottom). The consensus sequences of 9 individuals are given at the top of each alignment. There are 12 loci and 18 loci maintaining >= 2 amino acid alleles for the 9 crested ibis and the 9 little egrets, respectively. There are 1 loci and 6 loci maintaining >= 3 amino acid alleles for the 9 crested ibis and the 9 little egrets, respectively.

Figure S6

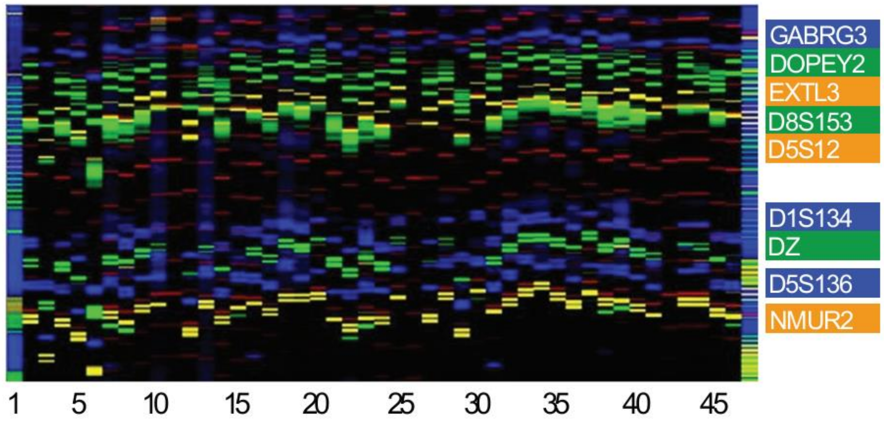

Figure S6. An example of STR genotyping. The PCR amplified products were separated by the ABI3730 Genetic Analyzer. Lanes 1 and 47 represent size markers and the remaining lanes depict PCR products of individual samples. Loci were labeled in multiple colors.

Figure S7

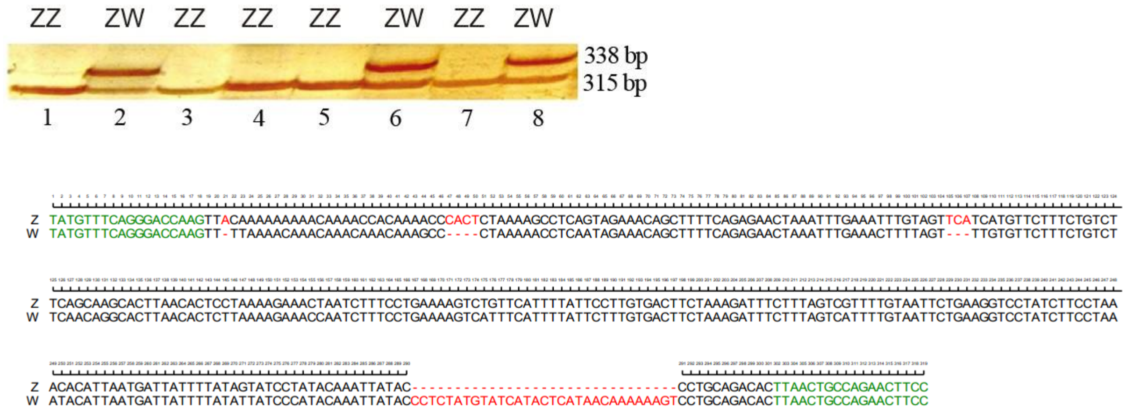

Figure S7 Sex discriminating locus of crested ibis. Electrophoresis is in the top panel. The sequence of the sex marker is in the bottom panel. The green bases represent primer sequences. The indels in Z and W chromosomes were marked in red.
